# Cycling infrastructure as a determinant of cycling for recreation and transportation in Montréal, Canada: a natural experiment using the longitudinal national population health survey

**DOI:** 10.1186/s12966-025-01767-y

**Published:** 2025-06-10

**Authors:** Stephanie A. Prince, Tyler Thomas, Philippe Apparicio, Lancelot Rodrigue, Christopher Jobson, Kathryn L. Walker, Gregory P. Butler, Rania Wasfi

**Affiliations:** 1 Centre for Surveillance and Applied Research, Health Promotion and Chronic Disease Prevention Branch, Public Health Agency of Canada, 785 Carling Ave, Ottawa, ON K1A 0K9 Canada; 2 School of Epidemiology and Public Health, Faculty of Medicine, University of Ottawa, Ottawa, Canada; 3 Public Health Sciences, Queen’s University, Kingston, Canada; 4 Department of Applied Geomatics, Université de Sherbrooke, Sherbrooke, Canada; 5 School of Urban Planning, McGill University, Montréal, Canada; 6 Interoperable Platforms and Product Management, Digital Transformation Branch, Health Canada, Ottawa, Canada; 7 Population Health, Faculty of Health Sciences, University of Ottawa, Ottawa, Canada; 8 Modelling Hub Division, Applied Public Health Sciences Directorate, Science and Policy Integration Branch, Public Health Agency of Canada, 200 René-Lévesque Blvd O, Montréal, QC H2Z 1X4 Canada

**Keywords:** Cycling, Recreation, Transportation, Natural experiment

## Abstract

**Background:**

Cycling is associated with numerous health benefits. Evidence suggests that new cycling infrastructure leads to increases in cycling, though studies of network-level changes are lacking. The objective of this study was to determine the longitudinal effect of cycling infrastructure on cycling engagement among adults living in Montréal, Canada.

**Methods:**

Using data from the National Population Health Survey (1994–2011), this study included adults who resided in the Montréal Census Metropolitan Area for a minimum of two survey cycles (*N* = 779). Outcomes included self-reported any cycling (transportation or recreation) and time in recreational cycling (minutes/week). Archival maps describing temporal changes in the cycling network for five-year intervals (1991–2011) were classified using the Canadian Bikeway Comfort and Safety Classification System (Can-BICS). Three cycling exposures were calculated from the centroid of each dissemination area: (1) distance to the nearest cycling path categorized by Can-BICS comfort-level (low, medium or high), (2) presence of cycle paths of each comfort level within distance thresholds (low = 321 m, medium = 623 m, high = 1790 m), and (3) density of cycle paths within a 1000 m buffer. Mixed effects logistic regression models estimated associations between cycling infrastructure and any cycling. Linear mixed effects models estimated associations between cycling infrastructure and time spent in recreational cycling.

**Results:**

Over the study period, low- and medium-comfort cycle paths were more prevalent than high-comfort paths and cycling for recreation was more common than cycling for transportation. Exposure to high-comfort paths within an acceptable distance (< 1790 m) was associated with higher odds of any cycling (aOR = 1.28, 95% CI: 1.00–1.63). Cumulative exposure to medium-comfort paths within an acceptable distance (< 623 m) was associated with greater time spent in recreational cycling (β = 0.09, 95% CI: 0.03–0.16). Gender-stratified analyses suggested that cumulative exposures to low- and medium-comfort infrastructure within distance thresholds was associated with time spent in recreational cycling (low: β = 0.06, 95% CI: 0.00–0.12, medium: β = 0.13, 95% CI: 0.04–0.22,) among women. No significant effects were observed for distance to the nearest cycling infrastructure for either outcome. Density was not examined in models due to low variation with most buffers having no cycling infrastructure.

**Conclusions:**

This research provides evidence that cycle paths, especially of higher comfort and safety, can promote cycling. Future work is needed to explore cumulative exposures to cycling infrastructure, taking into consideration connectivity of networks, integrated public transport, and accessibility to work.

**Supplementary Information:**

The online version contains supplementary material available at 10.1186/s12966-025-01767-y.

## Background

Regular physical activity (PA) is important for health and well-being [1–3]. PA is often classified into four domains reflecting the purpose of the activity including occupational/school, domestic (e.g., household chores, yard work, childcare), leisure (e.g., discretionary and recreation time including hobbies, sports, exercise), and transportation. PA that occurs in the transportation domain is often referred to as active transportation (AT; e.g., walking or cycling to get from one place to another). Among Canadian adults, 41.7% report using AT to commute to work or school, to access a bus stop, to go shopping or to visit friends [4]. Additionally, 6.2% of working Canadians (15 + years) reported using AT as their main mode of commuting to work (~ 1.1% cycled) [5, 6]. Adult Canadians engage in an average of 1.8 h of PA related to AT per week compared to 2.0 h of recreational PA, with PA, notwithstanding the type, being more prevalent among men than women [4]. People who engage in AT report higher levels of total PA, compared to those who do not [7], making it an important means to achieve recommended PA levels [8]. Consequently, increased AT [9–13] including cycling for transportation [14], have been shown to be associated with favourable health outcomes, as is the case for overall PA levels. A meta-analysis found that cycling 11.25 MET hours per week (~ 2 h per week) was associated with a 10% reduction in mortality, irrespective of an individual’s PA level [15]. Additionally, the dose-response curve showed the greatest gains were observed amongst those who had not previously cycled then started cycling [15]. This reduction in mortality has the potential to yield important and substantial population health benefits.

Features of cycling infrastructure which have been shown to be positively associated with cycling included: the presence of dedicated cycle routes or paths, separation of cycling from traffic, and proximity to a cycle path [16–18]; these associations, however, were mostly observed in cross-sectional studies; limiting causal inference. Some studies suggest that greater access to cycling infrastructure increases measured safety from traffic and injuries [19, 20], which trickles down into increased perceptions of safety by cyclists [21]. This latter point is crucial as the safer an individual feels while cycling, the more likely they are to cycle [22, 23]. Systematic review evidence from European countries, Australia, New Zealand, and the United States (US) suggests that interventions involving cycling infrastructure (e.g., opening new cycling lanes (i.e., designated areas within a roadway for cyclists) or paths (i.e., dedicated pathway separated from motorized traffic), expansion of a current network), result in increases in the number of cyclists and an increase in cycling [17, 24]. Conversely, intervention studies in Canada are scarce [25–28]. Overall, experimental and cross-sectional studies on built environments and PA amongst adults have generally found positive associations between cycling infrastructure and AT, with more mixed associations with recreational and total PA [29].

In Canada, open-source GIS data on cycling infrastructure at the national level was recently developed [16, 30, 31]. These sources quantify present-day cycling infrastructure but lack historical data to assess the interplay between changes in infrastructure and rates of cycling. The majority of Canadian studies have demonstrated cross-sectional associations between proximity and access to cycling infrastructure and cycling and PA [23, 32–35], and some have examined changes in cycling/PA following an infrastructure intervention using small pre-post studies [24–28]. To our knowledge, none have explored longitudinal changes in cycling infrastructure at the city-level and the impact of infrastructure changes on cycling rates from both a transportation and recreational perspective.

The objective of this study was to estimate the impact of exposure to cycling infrastructure on trajectories of cycling behaviour for recreation and transport in adults living in the Montréal Census Metropolitan Area (CMA), Québec, Canada. We hypothesized that higher comfort cycling infrastructure (e.g., dedicated paths separated from motorized traffic) and closer proximity to cycling infrastructure, would be positively related to the likelihood of cycling in adults, with those who gained closer proximity to higher comfort paths in their home neighbourhood more likely to maintain or increase their cycling over time.

## Methods

### National population health survey (NPHS)

The NPHS was a bi-annual population health survey that collected both cross-sectional and longitudinal data for the first three cycles (1994–1999), then became strictly longitudinal (collecting data from the same individual in each cycle) for the next six cycles, establishing a longitudinal cohort panel (1994–2011) [36, 37]. The survey excluded populations residing on Indian Reserves, those residing on Canadian Forces Bases, the territories, and residents of certain remote regions in Québec and Ontario [36, 37]. All survey responses were collected through computer-assisted interviewing which took place over the telephone or in person.

Our analysis was based on the longitudinal data from all nine cycles (1994/1995 to 2010/2011). The NPHS experienced a modest rate of annual attrition due to non-response, with an accumulated non-response rate of 7.5% of the total panel.

The study population included all respondents aged 18 years and older, who resided in the Montréal CMA for a minimum of two follow-up times with complete data on cycling for recreation and/or transport (*N* = 779). Entry into the cohort is defined as the first cycle in which respondents were 18 years or older and resided in the Montréal CMA. Those who moved within or out of the CMA and returned were included if they reported residing in the Montréal CMA for a minimum of two follow-up times.

### Dependent variable– cycling for recreation or transportation

The NPHS collected bi-annual self-reported data on cycling for recreation and transport. Participants were asked how many hours they usually spent (1) cycling to work, school or doing errands and (2) cycling for recreation for a typical week over the past three months. The main outcome of interest was ‘any cycling’ for transport or recreation (yes/no), which was derived by coding respondents who selected ‘none’ as ‘no’ for use and those that selected ‘greater than none’ for cycling to work or for leisure cycling as ‘yes’ for use. Additionally, duration of time (minutes per week) spent cycling for recreation was explored in secondary analyses (note there were too few individuals who reported cycling for transportation to explore duration of cycling for transportation). To convert the categorical responses to a continuous number, the responses were coded using the midpoint of the range for cycling for recreation (i.e., none = 0, 1–15 minutes = 7.5 minutes, 16 to 30 minutes = 23.5 minutes, 31–60 minutes = 46 minutes, and > 1 hour = 60 minutes).

### Montréal cycling infrastructure

Historical maps of the cycling infrastructure in the Montréal CMA were created for a previous study [38] in ArcGIS using archival maps and open-access data from the Cities of Montréal, Longueuil and Laval as well as Vélo Québec (a non-profit organization promoting cycling in Québec). These maps, which were generated for five-year intervals (1991, 1996, 2001, 2006, 2011), described the temporal changes in Montréal’s cycling network, demonstrating how the total length of cycling infrastructure doubled between 1991 and 2011 (from 270 km to 546 km) [38]. This data provided a unique opportunity to explore how the expansion of the cycling infrastructure in Montréal may have influenced cycling behaviour for recreation and transport over the same time period.

The GIS database not only spatialized the entire cycling network over multiple time periods, but it also recorded the type-specific cycling infrastructure which provided the opportunity to assess cycling infrastructure classified by comfort and safety using the Canadian Bikeway Comfort and Safety (Can-BICS) Classification System [31, 39]. The Can-BICS classifies cycling infrastructure into high-, medium- and low-comfort based on their design characteristics. High-comfort cycleways represent those that are low-stress including cycle tracks that are physically separated from traffic (e.g., use of bollards), local street bikeways (i.e., local streets with traffic calming where cyclists share the roadway with motor vehicles) and cycle-only off-street paths. Medium-comfort cycleways are generally represented by multi-use paths (i.e., pedestrians and cyclists share the paths) and are likely more frequented for recreational purposes. Low-comfort cycleways are high-stress and include painted bike lanes along busy roadways. The Can-BICS excludes cycling infrastructure with no dedicated lanes (i.e., sharrows) and with unpaved surfaces [39].

### Independent variable– Cycling infrastructure

Type-specific cycling infrastructure was classified using the Can-BICS into **high-comfort cycleways** (paths that are comfortable for most people including Type 1– off-street bicycle path and Type 2– on-street bicycle path with protection), **medium-comfort cycleways** (comfortable for some people, Type 7– multi-use path) and **low-comfort cycleways** (comfortable for few people, Type 3– on-street bicycle path without protection). Infrastructure that was not captured by the Can-BICS was considered non-conforming and excluded (Type 4– bike lanes which were sharrows, Type 5– shared bike lane and Type 6– park paths when they were unpaved). Visual inspections using Google Street View were used to verify the infrastructure presence and comfort level from the shapefiles (Supplementary materials 1).

Three cycling infrastructure exposure measures were calculated in ArcGIS using the Can-BICS classification: (1) the distance to the nearest cycling path for each comfort-level (low, medium or high), (2) the presence of cycling paths within acceptable distances for each comfort-level (low-comfort (321 m), medium-comfort (623 m) and high-comfort (1790 m)) and (3) the density of cycling infrastructure by comfort level. All measures were calculated from the centroid of the dissemination area (DA) of residence (proxied for the home location) for each respondent using 2006 Census Boundary files from Statistics Canada [40]. The DA is the smallest geographic area for which census data are available and generally includes 400 to 700 people. The NPHS data included six-digit postal codes which allowed respondents to be assigned to a DA and linked to their cycling infrastructure using the Postal Code Conversion File Plus (PCCF + V8A).

We employed an annual road network shapefile [41] in ArcGIS Pro 3.3 to calculate distances from each DA centroid to the nearest cycling infrastructure of each comfort level. To obtain the second exposure measure, we then converted these distances into binary variables denoting the presence of each comfort level infrastructure within acceptable distance thresholds. These selected thresholds were estimated from a 2009 Montréal survey of cyclists that identified average diversion length for low-comfort (321 m), medium-comfort (623 m) and high-comfort (1790 m) cycling infrastructure [42], which emphasize how people can travel longer distances to get into more comfort cycling paths.

The density of cycling infrastructure of each comfort level was calculated using a 1,000-meter buffer around the centroid of the DA of residence. An airline buffer was chosen over a network service area to allow for comparison with previous research [31]. Waterways and territory without cycling infrastructure data (around the periphery of the study area) were removed from the buffers. The total length of cycling infrastructure by comfort level was lastly divided by the total area of the cropped buffers, yielding a density of cycling infrastructure (in m/km^2^).

Table 1 provides the corresponding linkage years for cycling outcomes in the NPHS. All cycling path exposure variables were time varying (i.e. allowed to change year over year either by movement of participant or by expansion/introduction of the cycle paths). Cumulative exposure to cycling paths was also tested in analyses. Cumulative exposure to cycling paths was calculated as the cumulative time (in years) exposed to the different infrastructure types. Previous research on walking for utilitarian purposes using the same survey (NPHS), demonstrated that change in walking was positively associated with cumulative exposure to neighbourhood walkability [43, 44]. This indicated that change in travel behaviour does not happen immediately with the introduction of built environment supporting features but takes exposure time to see changes in travel behaviours.

**Table 1:** Cycling infrastructure and cycling outcome dataset years

Cycling infrastructure year	NPHS years (cycle #)
1991	1994/1995 (1)
1996	1996/1997 (2), 1998/1999 (3)
2001	2000/2001 (4), 2002/2003 (5), 2004/2005 (6)
2006	2006/2007 (7), 2008/2009 (8)
2011	2010/2011 (9)

### Covariates

We controlled for self-reported age centred at baseline, sex, immigrant status, season of data collection, education level (< post-secondary, post-secondary or greater), health-related quality of life using the Health Utility index (HUI) [45], and school or work status from the NPHS survey. Additionally, a “movers” variable was created by comparing the DA of residence between each cycle of the NPHS to denote whether the respondent had moved at all during the cohort. Neighbourhood walkability was assigned using the 2006 Canadian Active Living Environments (Can-ALE) [46] and neighbourhood deprivation was assigned using the 2006 Canadian Marginalization Index (CAN-Marg) [47]. Neighbourhood-level variables were linked by DA. Sex stratified analyses were undertaken to assess if the associations differed between men and women. While the NPHS only records sex, differences in cycling behaviour are predominantly gender-based (i.e., the reflection of social norms) rather than sex-based (i.e., biologically-driven) [47–53]. For the purpose of this paper, we have assumed that the NPHS data on respondents’ sex broadly corresponds to their gender identity. We acknowledge that the use of a unidimensional sex-based variable is a limitation as it does not capture the complexities of gender identity which is a multidimensional construct. All confounders were included as time-varying variables except for age, gender, immigrant status, walkability, and marginalization which were included as the value at baseline. All independent variables were assessed for multicollinearity using a correlation matrix and using the Variance Inflation Factor (VIF) for each variable in the models.

### Statistical analyses

Descriptive statistics including means or proportions and 95% confidence intervals (CIs) are presented for participant characteristics at baseline (the first cycle of entry into the cohort) including the cycling infrastructure exposure measures linked to their DA of residence and the cycling outcomes (Table 2).

We created a person-period dataset (one record per participant) for each survey cycle in which cycling for transport or leisure was available. We conducted an attrition analysis to compare the included sample with those who were excluded due to lost to follow-up to assess whether participants were lost at random, or we were left with a biased sample. We performed weighted t-tests and chi-square tests to determine if there was a statistical difference between the sample at a current cycle and those lost at that time. We compared cycling indicators as well as HUI score and age to get a sense of the overall well-being of those being lost and whether we were losing any cyclists.

To evaluate our binary outcome (having done any cycling or not), we used a mixed effects logistic regression model. This type of model accounts for subject-level heterogeneity seen in our data by estimating a random intercept and random slope. It separates within variance and between variance in the residual random error. Random effects from the subject-level allowed us to properly account for and model within and between variance, accounting for within person correlations in the observations, and reducing the error in the fixed effects estimates. To evaluate our continuous outcome (minutes of cycling per week), we specified a linear mixed effects model with identical covariates structure. We applied a log transformation to produce a more normally distributed outcome and obtain a better model fit. All models were sex-stratified in secondary analyses. Supplementary materials 2 provides the details for the model specification.

We conducted all analyses using R statistical software (version 4.4.1). To account for the complex sampling design of the NPHS, we used population survey weights to weight the sample appropriately. We normalized the weights to our sub-sample of individuals residing in the Montréal CMA to account for the fact that the weights were originally intended to be used on the full NPHS dataset across Canada. The new normalized weight vector appropriately had a mean of 1. Lastly, we used bootstrap weights (*n* = 500) for variance estimation for baseline descriptives.

## Results

### Descriptive statistics

At baseline our sample included 52% women, the majority having attained at least a post-secondary education (70%) and currently working or in school (76%). Additionally, 19% were immigrants, and the mean HUI score was 0.91, suggesting an overall healthy sample [54]. A total of 40% of the sample had moved during the study period. In terms of the outcome variables, a larger proportion of the sample had partaken in cycling for recreation (28%) compared to cycling for transportation (10%) (Table 2). Results of the attrition analyses (Supplementary materials 3) showed no difference between those retained in the sample and those who were lost to follow-up except for the HUI score (*p* = 0.001) at the second cycle. The absence of statistical differences between returning and lost respondents for all other tested variables across cycles, confirmed that attrition did not introduce statistically significant bias in our sample. Additionally, the introduction of new participants into the panel (i.e., moved into the Montréal CMA at different points in time), helped maintain the randomness of loss due to follow-up.

**Fig. 1:**
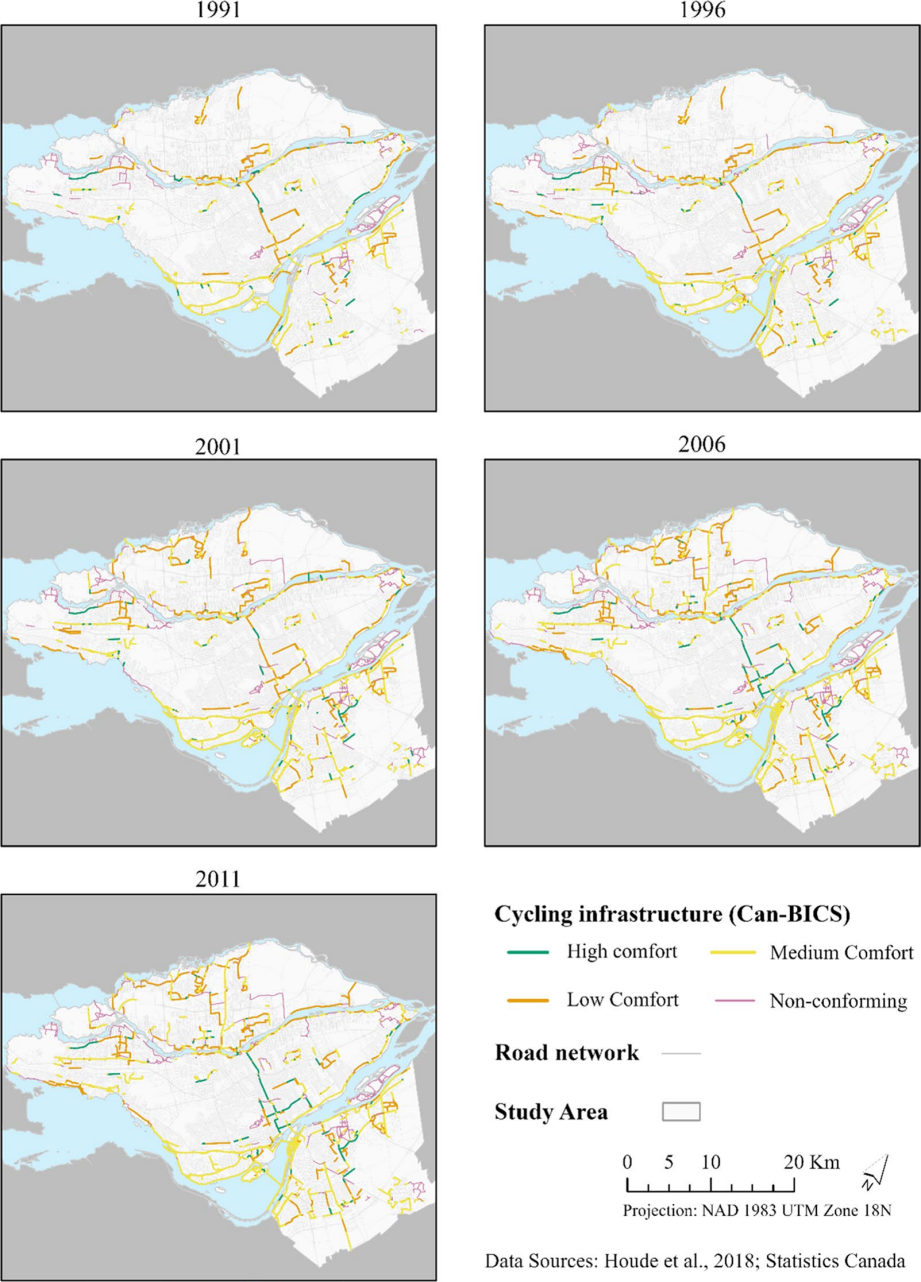
Cycling infrastructure expansion in the Montréal Census Metropolitan Area, Québec, 1991 to 2016

With regards to the exposure variables, cycling infrastructure in the Montréal CMA expanded in different parts of the CMA from 1991 to 2011, increasing the prevalence of cycling paths of all comfort and safety levels; with different degrees based on Can-BICS classification (Fig. 1).

**Fig. 2:**
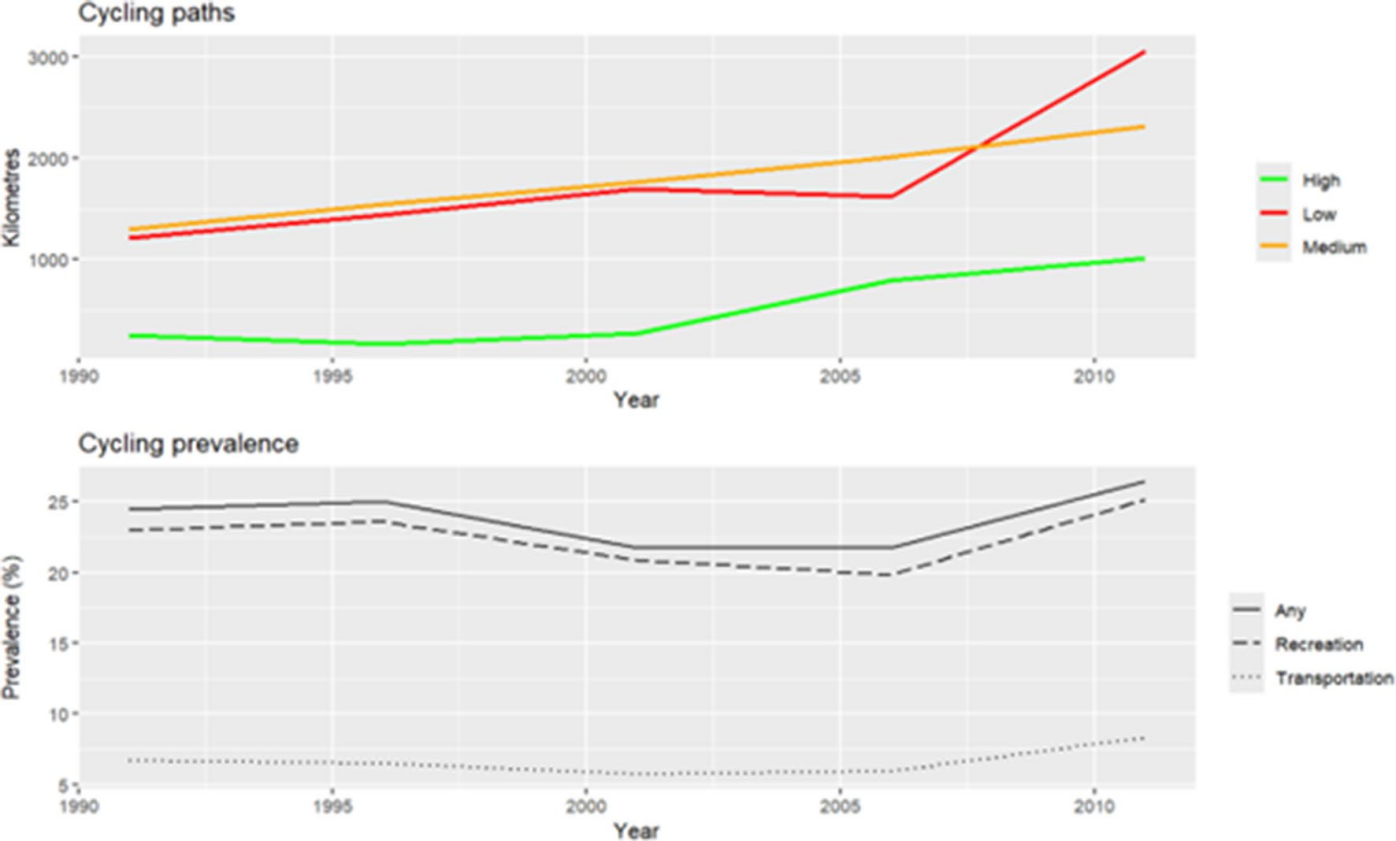
Cycling infrastructure length (**a**) and prevalence of cycling (**b**) over time, the Montréal Census Metropolitan Area, Québec

Over the entire study period, low- and medium-comfort cycle paths were more prevalent than high-comfort paths and cycling for recreation was more common than cycling for transportation (Fig. 2). Supplementary materials 4 provides the km of cycling infrastructure for each year by Can-BICS category. We found that 34% of the population sample were exposed to high comfort paths within the pre-defined ‘acceptable diversion distance’ threshold of 1790 m, 15% exposed to medium comfort within 623 m, and 9% to low-comfort within 321 m at baseline. For comparison, participant end-of-study values included 49% exposed to high-comfort within 1790 m, 26% exposed to medium-comfort within 623 m and 16% to low comfort within 321 m. Density of cycle paths around the home location were ultimately not included in the regression models due to low variation with most buffers created around DAs having no cycling infrastructure (mode = 0).

**Table 2:** Baseline participant characteristics

Characteristics		Unweighted *n*	Weighted mean or % (95% CI)
Individual characteristics			
**Mean age**,** years**		779	35.2 (34.2, 36.2)
**Gender**			
Men		344	48% (44%, 51%)
Women		435	52% (49%, 56%)
**Education level**			
Post-secondary or greater		488	70% (65%, 75%)
Less than post-secondary		284	30% (25%, 35%)
**Immigrant status**			
Yes		144	19% (14%, 23%)
No		634	81% (77%, 86%)
**School and work school status**			
Currently employed or in school		505	76% (72%, 80%)
Not in school or work		173	24% (20%, 28%)
**Moved during the study**			
Yes		331	40% (34%, 46%)
No		448	60% (54%, 66%)
**Health Utility Index**,** score**		779	0.91 (0.90, 0.93)
**Season of data collection**			
Spring		173	24% (20%, 28%)
Summer		195	23% (19%, 28%)
Fall		188	26% (21%, 31%)
Winter		205	26% (22%, 31%)
**Cycling for transportation**			
Yes		66	10% (7%, 12%)
No		660	90% (88%, 93%)
**Cycling for transportation**,** min/day**		779	20 (12, 28)
**Cycling for recreation**			
Yes		193	28% (23%, 33%)
No		535	72% (67%, 77%)
**Cycling for recreation**,** min/day**		779	11 (9, 13)
**Home DA-level characteristics of participants**			
**Marginalization Index**,** score**		779	3.61 (3.49, 3.73)
**Walkability Index**,** score**		779	2.64 (2.39, 2.89)
**Cycling infrastructure closest distance**			
High-comfort, metres		779	2842 (2465, 3219)
Medium-comfort, metres		779	1757 (1552, 1962)
Low-comfort, metres		779	1909 (1634, 2184)
**Cycling infrastructure distance thresholds**			
High comfort (< 1790 m), yes		779	34% (24%, 43%)
Medium comfort (< 623 m), yes		779	15% (11%, 20%)
Low comfort (< 321 m), yes		779	9% (5%, 14%)
**Cycling infrastructure density**			
High-comfort, m/km^2^		779	59.1 (30.2, 88.0)
Medium-comfort, m/km^2^		779	201.1 (155.3, 246.9)
Low-comfort, m/km^2^		779	234.7 (162.9, 306.6)

### Mixed effects model results

We evaluated the effect of exposure to cycling infrastructure (time varying distance thresholds and cumulative years at distance thresholds) on engagement in any cycling and recreational cycling time in the Montréal CMA (Tables 3, 4, 5, 6, 7 and 8). We found that having access to high-comfort paths within the threshold distance (1790 m) was associated with higher odds of any cycling (aOR = 1.28, 95% CI: 1.00–1.63) (Table 3). When considering a cumulative exposure, we found that cumulative exposure to medium-comfort pathways within the threshold distance (623 m) was associated with greater time spent in recreational cycling (β = 0.09, 95% CI: 0.03–0.16). Cumulative exposure to high-comfort pathways within the threshold distance (1790 m), approached statistical significance (β = 0.04, 95% CI: 0.00–0.09). (Table 7).

**Table 3:** Associations between access to time varying cycling infrastructure within distance thresholds and any cycling (*N* = 776)

Fixed Effects	Unadjusted				Adjusted			
	OR	95% CI	SD	*p*-value	OR	95% CI	SD	*p*-value
Time in years	0.95	0.91, 0.99	0.02	0.0213	0.91	0.87, 0.96	0.02	0.0002
High-comfort threshold (< 1790 m)	**1.23**	**0.97**,** 1.56**	**0.12**	**0.0896**	**1.28**	**1.00**,** 1.63**	**0.12**	**0.0458**
Medium-comfort threshold (< 623 m)	0.78	0.57, 1.07	0.16	0.1248	0.87	0.63, 1.19	0.16	0.3701
Low-comfort threshold (< 321 m)	1.31	0.90, 1.89	0.19	0.1585	1.10	0.75, 1.62	0.20	0.6299
Baseline age					0.96	0.95, 0.97	0.01	0.0000
Men (ref. Women)					1.65	1.21, 2.25	0.16	0.0014
Health Utility Index					2.56	1.05, 6.25	0.46	0.0386
Post-secondary education (ref. < post-secondary)					1.21	0.86, 1.71	0.18	0.2777
Walkability Index					0.98	0.91, 1.04	0.03	0.4712
Immigrant (ref. Non-immigrants)					0.64	0.42, 0.96	0.21	0.0331
Working/in school (ref. Not working/in school)					1.33	0.98, 1.79	0.15	0.0663
Marginalization Index					0.82	0.69, 0.98	0.09	0.0274
Movers (ref. Non-movers)					0.83	0.64, 1.07	0.13	0.1471
Season (ref. Fall season)								
Spring season					0.74	0.52, 1.05	0.18	0.0924
Summer season					1.46	1.05, 2.03	0.17	0.0243
Winter season					0.17	0.11, 0.25	0.20	0.0000

Random effects (adjusted model): Random intercept variance = 1.84, random slope variance = 0.05. CI = confidence interval, OR = odds ratio, SD = standard deviation

**Table 4:** Associations between cumulative years of exposure to cycling infrastructure within distance thresholds and any cycling (*N* = 776)

Fixed Effects	Unadjusted				Adjusted			
	OR	95% CI	SD	*p*-value	OR	95% CI	SD	*p*-value
Time in years	1.14	1.03, 1.27	0.05	0.013	0.96	0.86, 1.07	0.06	0.4293
High-comfort threshold (< 1790 m)	**0.91**	**0.85**,** 0.97**	**0.03**	**0.004**	0.99	0.92, 1.06	0.03	0.6851
Medium-comfort threshold (< 623 m)	**0.88**	**0.80**,** 0.96**	**0.05**	**0.005**	0.96	0.87, 1.06	0.05	0.4363
Low-comfort threshold (< 321 m)	**0.90**	**0.85**,** 0.96**	**0.03**	**0.001**	0.97	0.92, 1.04	0.03	0.4063
Baseline age					0.96	0.95, 0.98	0.01	0.0000
Men (ref. Women)					1.65	1.21, 2.24	0.16	0.0015
Health Utility Index					2.49	1.02, 6.07	0.45	0.0450
Post-secondary education (ref. < post-secondary)					1.19	0.84, 1.69	0.18	0.3144
Walkability Index					0.98	0.92, 1.05	0.03	0.5767
Immigrant (ref. Non-immigrants)					0.63	0.42, 0.96	0.21	0.0315
Working/in school (ref. Not working/in school)					1.32	0.98, 1.78	0.15	0.0716
Marginalization Index					0.83	0.69, 0.99	0.09	0.0353
Movers (ref. Non-movers)					0.83	0.64, 1.07	0.13	0.1488
Season (ref. Fall season)								
Spring season					0.76	0.53, 1.08	0.18	0.1203
Summer season					1.49	1.07, 2.07	0.17	0.0189
Winter season					0.17	0.11, 0.25	0.20	0.0000

Random effects (adjusted model): Random intercept variance = 1.85, random slope variance = 0.05. CI = confidence interval, OR = odds ratio, SD = standard deviation

**Table 5:** Associations between shortest distance to type of time varying cycling infrastructure from centroid of dissemination area and any cycling (*N* = 776)

Fixed Effects	Unadjusted				Adjusted			
	OR	95% CI	SD	*p*-value	OR	95% CI	SD	*p*-value
Time in years	0.96	0.91, 1.00	0.02	0.0562	0.92	0.87, 0.96	0.02	0.0003
High-comfort threshold (< 1790 m)	0.97	0.91, 1.04	0.03	0.3937	0.96	0.90, 1.03	0.03	0.2215
Medium-comfort threshold (< 623 m)	1.07	0.96, 1.19	0.05	0.2262	1.02	0.92, 1.14	0.05	0.6825
Low-comfort threshold (< 321 m)	1.00	0.92, 1.09	0.04	0.9862	1.00	0.92, 1.09	0.04	0.9589
Baseline age					0.96	0.95, 0.97	0.01	0.0000
Men (ref. Women)					1.67	1.22, 2.27	0.16	0.0012
Health Utility Index					2.48	1.02, 6.06	0.46	0.0458
Post-secondary education (ref. < post-secondary)					1.19	0.84, 1.69	0.18	0.3249
Walkability Index					0.97	0.91, 1.05	0.04	0.4745
Immigrant (ref. Non-immigrants)					0.62	0.41, 0.94	0.21	0.0246
Working/in school (ref. Not working/in school)					1.33	0.98, 1.80	0.15	0.0667
Marginalization Index					0.81	0.68, 0.97	0.09	0.0225
Movers (ref. Non-movers)					0.82	0.64, 1.07	0.13	0.1469
Season (ref. Fall season)								
Spring season					0.75	0.53, 1.07	0.18	0.1079
Summer season					1.48	1.06, 2.07	0.17	0.0198
Winter season					0.17	0.11, 0.25	0.20	0.0000

Random effects (adjusted model): Random intercept variance = 1.92, random slope variance = 0.05. CI = confidence interval, OR = odds ratio, SD = standard deviation

**Table 6:** Associations between access to time varying cycling infrastructure within distance thresholds and log minutes per week of recreational cycling (*N* = 379)

Fixed Effects	Unadjusted				Adjusted			
	Coef.	95% CI	SD	*p*-value	Coef.	95% CI	SD	*p*-value
Time in years	0.02	-0.02, 0.06	0.02	0.3226	0.03	0.00, 0.07	0.02	0.0847
High-comfort threshold (< 1790 m)	-0.02	-0.21, 0.16	0.09	0.8234	-0.01	-0.20, 0.18	0.10	0.9121
Medium-comfort threshold (< 623 m)	-0.07	-0.30, 0.16	0.12	0.5585	-0.09	-0.32, 0.15	0.12	0.4754
Low-comfort threshold (< 321 m)	-0.01	-0.30, 0.28	0.15	0.9456	-0.02	-0.32, 0.27	0.15	0.8775
Baseline age					0.00	-0.01, 0.01	0.00	0.4724
Men (ref. Women)					0.23	0.01, 0.46	0.11	0.0372
Health Utility Index					0.68	-0.05, 1.42	0.37	0.0690
Post-secondary education (ref. < post-secondary)					-0.08	-0.35, 0.18	0.13	0.5385
Walkability Index					0.09	0.04, 0.14	0.03	0.0003
Immigrant (ref. Non-immigrants)					0.38	0.02, 0.73	0.18	0.0372
Working/in school (ref. Not working/in school)					-0.35	-0.59, -0.10	0.12	0.0051
Marginalization Index					-0.11	-0.24, 0.03	0.07	0.1224
Movers (ref. Non-movers)					0.20	0.00, 0.40	0.10	0.0458
Season (ref. Fall season)								
Spring season					-0.28	-0.55, -0.02	0.14	0.0356
Summer season					-0.02	-0.26, 0.21	0.12	0.8541
Winter season					-0.31	-0.64, 0.02	0.17	0.0636

Random effects (adjusted model): Random intercept variance = 1.23, random slope variance = 0.03. CI = confidence interval, SD = standard deviation

**Table 7:** Associations between cumulative years of exposure to cycling infrastructure within a distance threshold and log minutes per week of recreational cycling (*N* = 379)

Fixed Effects	Unadjusted				Adjusted			
	Coef.	95% CI	SD	*p*-value	Coef.	95% CI	SD	*p*-value
Time in years	-0.05	-0.12, 0.02	0.04	0.1813	-0.03	-0.11, 0.04	0.04	0.3521
High-comfort threshold (< 1790 m)	**0.04**	**0.00**,** 0.08**	**0.02**	**0.0792**	**0.04**	**0.00**,** 0.09**	**0.02**	**0.0674**
Medium-comfort threshold (< 623 m)	**0.07**	**0.01**,** 0.14**	**0.03**	**0.0157**	**0.09**	**0.03**,** 0.16**	**0.03**	**0.0060**
Low-comfort threshold (< 321 m)	0.03	-0.01, 0.07	0.02	0.1090	0.03	-0.01, 0.07	0.02	0.1621
Baseline age					0.00	-0.01, 0.01	0.01	0.9386
Men (ref. Women)					0.27	0.05, 0.48	0.11	0.0173
Health Utility Index					0.66	-0.08, 1.39	0.37	0.0790
Post-secondary education (ref. < post-secondary)					-0.08	-0.34, 0.18	0.13	0.5438
Walkability Index					0.09	0.04, 0.14	0.03	0.0002
Immigrant (ref. Non-immigrants)					0.34	-0.01, 0.69	0.18	0.0546
Working/in school (ref. Not working/in school)					-0.34	-0.59, -0.10	0.12	0.0053
Marginalization Index					-0.10	-0.24, 0.03	0.07	0.1266
Movers (ref. Non-movers)					0.20	0.00, 0.40	0.10	0.0493
Season (ref. Fall season)								
Spring season					-0.31	-0.58, -0.05	0.14	0.0206
Summer season					-0.05	-0.28, 0.19	0.12	0.7024
Winter season					-0.34	-0.67, -0.01	0.17	0.0417

Random effects (adjusted model): Random intercept variance = 1.23, random slope variance = 0.03. CI = confidence interval, SD = standard deviation

**Table 8:** Associations between shortest distance to time varying cycling infrastructure and log minutes per week of recreational cycling (*N* = 379)

Fixed Effects	Unadjusted				Adjusted			
	Coef.	95% CI	SD	*p*-value	Coef.	95% CI	SD	*p*-value
Time in years	0.01	-0.03, 0.05	0.0	0.5708	0.03	-0.01, 0.07	0.02	0.1363
High-comfort threshold (< 1790 m)	-0.01	-0.06, 0.03	0.03	0.5601	-0.02	-0.07, 0.04	0.03	0.5488
Medium-comfort threshold (< 623 m)	0.02	-0.06, 0.09	0.04	0.6760	0.02	-0.05, 0.09	0.04	0.5467
Low-comfort threshold (< 321 m)	-0.04	-0.10, 0.02	0.03	0.2045	-0.01	-0.08, 0.05	0.03	0.6648
Baseline age					0.00	-0.01, 0.01	0.00	0.4525
Men (ref. Women)					0.24	0.02, 0.47	0.11	0.0307
Health Utility Index					0.69	-0.04, 1.43	0.37	0.0646
Post-secondary education (ref. < post-secondary)					-0.08	-0.34, 0.18	0.13	0.5314
Walkability Index					0.09	0.04, 0.14	0.03	0.0009
Immigrant (ref. Non-immigrants)					0.38	0.03, 0.73	0.18	0.0344
Working/in school (ref. Not working/in school)					-0.34	-0.58, -0.10	0.12	0.0063
Marginalization Index					-0.11	-0.25, 0.02	0.07	0.1088
Movers (ref. Non-movers)					0.20	0.01, 0.40	0.10	0.0442
Season (ref. Fall season)								
Spring season					-0.28	-0.55, -0.02	0.14	0.0360
Summer season					-0.02	-0.25, 0.22	0.12	0.8842
Winter season					-0.31	-0.64, 0.02	0.17	0.0636

Random effects (adjusted model): Random intercept variance = 1.25, random slope variance = 0.03. CI = confidence interval, SD = standard deviation

### Sex stratified model results

Supplementary materials 5-16– present results from the sex-stratified analyses. Among women, cumulative exposure to medium- and low-comfort cycling infrastructure within distance thresholds were significantly associated with time spent in recreational cycling (medium: β = 0.13, 95% CI: 0.04–0.22, low: β = 0.06, 95% CI: 0.00–0.12). Additionally, high-comfort associations approached statistical significance (β = 0.06, 95% CI: 0.00–0.12) (Supplementary material 14 ). We did not detect a significant effect for closest distance of cycling infrastructure for any cycling or time spent in recreational cycling.

## Discussion

This study is the first to explore the associations between changes to an entire cycling network and self-reported population-level cycling for transportation and recreation in a Canadian metropolitan area. We found that exposure to high-comfort cycling infrastructure within a predefined acceptable distance threshold was associated with a 28% greater likelihood of engagement in cycling for recreation or transportation. Additionally, cumulative years of exposure to medium-comfort cycling infrastructure within an acceptable distance threshold was associated with greater time spent in recreational cycling. The effects for cumulative exposure to cycling infrastructure within acceptable thresholds for low- and medium-comfort (high approached significance) and time spent recreational cycling were significant among females, but not males. No significant associations were observed when cycling infrastructure was explored as either time varying closest distance or density within 1 km buffer around the centroid of the DA.

Use of pre-existing data is a means to study natural experiments when the researchers have no control over the intervention or its timing, and these methods can be applied to other longitudinal data sets with cycling content. In the case of the present study, it provided the means to examine the influence of the expansion of the Montréal cycling network over a 20-year period. We are not aware of any other studies that have used a repeated measures design over such a long period to evaluate the effectiveness of cycle paths for changing cycling behaviour. A recent Delphi survey found that both researchers and knowledge users feel that the evaluation of natural experiments continues to be a top priority gap in research on built environments and physical activity [55].

We also explored three different operationalizations of the cycling infrastructure exposure, looking at time varying and cumulative time exposed to cycle paths within distance thresholds and time varying closest distance to cycle paths. The use of a cumulative exposure in addition to time varying exposures, adjusted for the fact that people may need to be exposed to new infrastructure long enough to make a change to behaviour and see a change in social norms [43, 56, 57]. The analysis was further strengthened by the application of the Can-BICS classification system to categorize cycle paths by their comfort and safety levels. Unfortunately, several of the cycling paths were non-Can-BICS conforming and were excluded from our analysis.

Although cycling infrastructure in the Montréal CMA increased between 1991 and 2011, with low- and medium-comfort bikeways representing the majority of infrastructure over time, most DAs remained exposed to little-to-no infrastructure. Overall prevalence of cycling increased over this time period, however, cycling for recreation remained higher than cycling for transportation. The largest expansion in cycle infrastructure occurred between 2006 and 2011, towards the end of the NPHS survey collection. This can partially explain why we have not seen an overall statistically significant increase in cycling behaviour; regardless of the cycling network comfort level.

The 2007 Montréal Transportation Plan [58] included a program to double the number of bike lanes in Montréal from 400 km to 800 km, with the majority of the proposed lanes being designed for recreational use. Unfortunately, while there was growth, in 2011 the cycling network represented only 10.7% of all potentially cyclable roads in Montréal and the network had limited connectivity [38]. In present day, the city of Montréal is considered one of the most bike-friendly cities in Canada with its cycle paths, bicycle share programme and winter maintenance [59]. Montréal is currently developing an Express Bike Network (Réseau Express Vélo [REV]) which will include 191 km of connected, physically separated cycling lanes accessible year round [60]. Recent qualitative data from Montréal suggests that residents are generally satisfied with the increase in high-comfort cycling infrastructure from the REV including its enhanced safety and family-friendliness [61]. The City has a goal to increase its cycling modal share to 15% by 2027 [62]. Examining change in cycling behaviour in recent years with all these expansions is important.

Our findings confirm evidence from systematic reviews on intervention studies, which suggests a generally positive association between new cycle paths or the expansion of networks and cycling levels [24, 63]. Most of these interventional research studies, however, have focused on the addition of single cycle paths, rather than evaluating change to a whole cycling network [64]. Our study also confirmed findings from a recent longitudinal study in Hamilton, Ontario which found that the creation of concrete-barrier separated cycle tracks (i.e., higher comfort) were more positively associated with cycling compared to painted and plastic barrier lanes (i.e., lower comfort) [65].

Studies that have explored cycling behaviours as outcomes found smaller effect sizes than those that explored infrastructure usage (e.g., direct observations). Most studies have used a pre-post design to explore changes in cycling before and after the new cycling paths were created. A recent study [28] confirms findings demonstrating no statistically significant associations between cycling and proximity to cycling infrastructure. The study [28] evaluated the impact of a 15 km ‘all ages and abilities’ cycling network in Victoria, Canada and found no significant change in self-reported previous year cycling over the intervention period (2016–2021) among those living closer rather than farther (> 500 m) from the new cycle paths. The authors mentioned the potential impact of the COVID-19 pandemic, as well as the cycling outcome used on the outcome of the study. In contrast, a three-year pre-post evaluation of a new urban greenway in Vancouver, Canada found a 251% increase in cycling trips in the experimental group (living within 300 m) compared to a control group (living father away) [27].

Expansions of cycling networks are crucial to promote increased access to destinations by cycling and usage. That being said, not all cycling infrastructure offers the same level of safety and comfort. Indeed, access to dedicated cycling infrastructure is associated with higher perceived safety for cycling compared to non-dedicated lanes [20]. Off-street and separated cycle paths are associated with fewer cyclist-vehicle interactions [66]. This is particularly important when considering how cyclists’ safety, both objectively measured and perceived, plays a key role in promoting cycling engagement [39]. The Can-BICS integration of cyclists’ safety and comfort [39] within its classification system allows to assess the quality of cycling infrastructure in addition to its quantity. Previous work has shown that Can-BICS classified cycling infrastructure, especially of higher safety and comfort, is positively associated with cycling and active transportation [31] and [73]. Findings from our study also suggest that exposure to high- and medium-comfort infrastructure are particularly important for promoting engagement in any cycling, and greater time spent in recreational cycling, respectively. Previous work in Québec City also suggests that cyclists prefer recreational paths over painted bike lanes for their commute [67].

The present study only found significant effects when assessing access to cycling infrastructure within distance thresholds based on the median acceptable diversion length of each infrastructure type [42]. Our results suggest that continuous exposure to higher comfort paths within an acceptable distance may positively influence the likelihood of cycling. This can be attributed to the greater level of safety and comfort of such infrastructure which entice cyclists to travel further to access them [68]. The lack of significance for the closest distance could be attributed to the limited extent of the Montréal cycling network during the period of study and our relatively small sample size. The contrasting results observed between the cumulative exposure and time varying models may be reflective of the existence of complementary short-term and long-term pathways through which changes in cycling infrastructure can impact cycling engagement and frequency. The short-term impacts of new cycling infrastructure might be witnessed through the rerouting of existing cycling trips to the improved infrastructure (i.e., route convergence), while the long-term cumulative effects might be understood as the normalization and popularization of cycling due to the continual exposure to improved cycling infrastructure (i.e., induced demand).

Results from this study suggest that greater cumulative exposure to cycle paths within their respective diversion distances, promoted more time spent cycling for recreation among women, but not men. This finding is coherent with previous studies which have hinted to a stronger relationship between cycling infrastructure and cycling frequency for women compared to men [34, 69, 70], especially cycling infrastructure of higher comfort/safety and cycling among women [52, 53, 69]. Such differences have been observed through gendered effects of perceptions of the convenience of cycling on utilitarian cycling [51] as well as through greater preference for off-road paths and safer cycling facilities for women compared to men [49, 50]. Beyond simple binary comparisons, differences between women and men in the factors influencing cycling behaviour (e.g., perceived safety, convenience and comfort) are compounded by gendered social norms [48]. While we assessed binary differences, we recognize that the cumulative effect of cycling infrastructure on cycling behaviour is likely to vary amongst women and men along with other key sociodemographic and attitudinal characteristics.

### Limitations

One of the main limitations of the study is the potential for the misclassification of infrastructure based on census year rather than survey year. It’s possible that some of the infrastructure appeared between census years. Additionally, while the analysis adjusted for neighbourhood social deprivation and walkability, these data were from 2006, and may have also resulted in some misclassification. The threshold distances applied in the analyses were identified from a 2009 survey of Montréal cyclists [42], but may not represent acceptable distances for access for all. Previous studies have explored shorter distances [27, 28], but this was used for all cycle paths regardless of comfort or safety. Previous research has also suggested that cyclists may opt to travel an additional 10% or more of the trip distance in order to access cycling infrastructure [71]. The study assessed presence of infrastructure, but did not assess the connectivity of the cycling network. Future research is needed to explore the effect of cycling infrastructure connectivity on the use of cycling [72]. We were unable to explore duration of cycling for transportation as too few respondents reported cycling for this purpose. Additionally, although we attempted to control for important confounding factors for which we had data access, there is the potential for residual unaccounted confounding including potential concurrent investments in motor vehicle and public transit infrastructure. Finally, our sample size may have been too small to detect associations given the low density of cycling infrastructure across most DAs. As more cycling infrastructure is reported [30] the relationships between infrastructure and cycling behaviour should become more clear.

## Conclusions

Exposure to cycling infrastructure within acceptable distances, especially of higher comfort and safety, is associated with increased cycling engagement. Additionally, among women, cumulative exposure within distance thresholds is associated with more time spent in recreational cycling. This research further strengthens the evidence in Canada, that cycle paths, especially of higher comfort and safety, can promote cycling. We also highlight differing effects of new cycling infrastructure across short-term and long-term periods. Given the continued growth of the cycling network in Montréal, Canada, future work will be needed to explore the ongoing influence of this expansion with consideration of cumulative exposure.

## Electronic supplementary material

Below is the link to the electronic supplementary material.

Supplementary Material 1

Supplementary Material 2

Supplementary Material 3

Supplementary Material 4

Supplementary Material 5

Supplementary Material 6

Supplementary Material 7

Supplementary Material 8

Supplementary Material 9

Supplementary Material 10

Supplementary Material 11

Supplementary Material 12

Supplementary Material 13

Supplementary Material 14

Supplementary Material 15

Supplementary Material 16

## Data Availability

The data sets analyzed for this study are available through the Research Data Centers (RDC) Program at Statistics Canada (https://www.statcan.gc.ca/en/microdata/data-centres).

## Abbreviations

AT: Active transportation
Can-ALE: Canadian Active Living Environments
Can-BICS: Canadian Bikeway Comfort and Safety (Can–BICS)Classification System
CAN-Marg: Canadian Marginalization Index
CI: Confidence interval
CMA: Census Metropolitan Area
DA: Dissemination area
HUI: Health Utility Index
MET: Metabolic equivalent of task
NPHS: National Population Health Survey
OR: Odds ratio
PA: Physical activity
REV: Réseau Express Vélo
SD: Standard deviation
US: United States
