# Supplementary material for: Cycling infrastructure as a determinant of cycling for recreation and transportation in Montréal, Canada: a natural experiment using the longitudinal national population health survey
Source: Int J Behav Nutr Phys Act. 2025 Jun 10;22:71. doi: 10.1186/s12966-025-01767-y (PMC12153112; doi:10.1186/s12966-025-01767-y)
Supplement: Supplementary file 1 — Supplementary Material 1 [file 12966_2025_1767_MOESM13_ESM.pdf]

Supplementary material 13. Associations between cumulative years of exposure to cycling infrastructure within distance thresholds and log minutes per week of recreational cycling in men (N=187)

Fixed Effects	Unadjusted				Adjusted			
	Coef.	95% CI	SD	p-value	Coef.	95% CI	SD	p-value
Time	-0.02	-0.11, 0.08	0.05	0.7009	0.01	-0.10, 0.11	0.05	0.9135
High Comfort Threshold (years)	0.02	-0.05, 0.08	0.03	0.6115	0.00	-0.06, 0.07	0.03	0.8807
Medium Comfort Threshold (years)	0.06	-0.02, 0.15	0.04	0.1358	0.04	-0.05, 0.13	0.05	0.4145
Low Comfort Threshold (years)	0.00	-0.05, 0.05	0.02	0.9794	-0.01	-0.07, 0.04	0.03	0.6287
Baseline age					0.00	-0.01, 0.02	0.01	0.7131
Health Utility Index					0.56	-0.35, 1.46	0.46	0.2260
Education					0.10	-0.28, 0.47	0.19	0.6140
Walkability Index					0.07	0.00, 0.13	0.03	0.0590
Immigrant					0.34	-0.13, 0.81	0.24	0.1574
Work/School					-0.20	-0.56, 0.17	0.19	0.2830
Marginalization Index					-0.11	-0.31, 0.09	0.10	0.2683
Movers					0.30	0.02, 0.58	0.14	0.0364
Spring season					-0.27	-0.65, 0.12	0.20	0.1735
Summer season					0.13	-0.21, 0.47	0.17	0.4610
Winter season					-0.45	-0.92, 0.01	0.24	0.0548

Random effects (adjusted model): Random intercept SD = 1.13, random slope SD = 0.15.

CI = confidence interval, SD = standard deviation
