# Supplementary material for: Cycling infrastructure as a determinant of cycling for recreation and transportation in Montréal, Canada: a natural experiment using the longitudinal national population health survey
Source: Int J Behav Nutr Phys Act. 2025 Jun 10;22:71. doi: 10.1186/s12966-025-01767-y (PMC12153112; doi:10.1186/s12966-025-01767-y)
Supplement: Supplementary file 2 — Supplementary Material 2 [file 12966_2025_1767_MOESM14_ESM.pdf]

Supplementary material 14. Associations between cumulative years of exposure to cycling infrastructure within distance thresholds and log minutes per week of recreational cycling in women (N=192)

Fixed Effects	Unadjusted				Adjusted			
	Coef.	95% CI	SD	p-value	Coef.	95% CI	SD	p-value
Time	-0.08	-0.19, 0.02	0.05	0.0968	-0.05	-0.16, 0.06	0.05	0.3475
High Comfort Threshold (<1790m)	**0.06**	**0.00, 0.13**	**0.03**	**0.0424**	0.06	0.00, 0.12	0.03	0.0557
Medium Comfort Threshold (<623m)	**0.10**	**0.01, 0.19**	**0.04**	**0.0218**	**0.13**	**0.04, 0.22**	**0.05**	**0.0067**
Low Comfort Threshold (<321m)	**0.07**	**0.01, 0.12**	**0.03**	**0.0246**	**0.06**	**0.00, 0.12**	**0.03**	**0.0473**
Baseline age					0.00	-0.01, 0.01	0.01	0.9838
Health Utility Index					0.86	-0.39, 2.12	0.64	0.1760
Education					-0.23	-0.59, 0.13	0.18	0.2152
Walkability Index					0.12	0.04, 0.19	0.04	0.0015
Immigrant					0.34	-0.20, 0.88	0.27	0.2143
Work/School					-0.44	-0.77, -0.11	0.17	0.0090
Marginalization Index					-0.11	-0.29, 0.07	0.09	0.2365
Movers					0.09	-0.19, 0.37	0.14	0.5366
Spring season					-0.34	-0.71, 0.03	0.19	0.0717
Summer season					-0.16	-0.48, 0.17	0.17	0.3436
Winter season					-0.16	-0.64, 0.32	0.24	0.5080

Random effects (adjusted model): Random intercept SD = 1.03, random slope SD = 0.18.

CI = confidence interval, SD = standard deviation
