# Supplementary material for: Cycling infrastructure as a determinant of cycling for recreation and transportation in Montréal, Canada: a natural experiment using the longitudinal national population health survey
Source: Int J Behav Nutr Phys Act. 2025 Jun 10;22:71. doi: 10.1186/s12966-025-01767-y (PMC12153112; doi:10.1186/s12966-025-01767-y)
Supplement: Supplementary file 3 — Supplementary Material 3 [file 12966_2025_1767_MOESM16_ESM.pdf]

Supplementary material 16. Associations between shortest distance to time varying type of cycling infrastructure from centroid of dissemination area and minutes per week of recreational cycling in women (N=192)

**Fixed Effects**	**Unadjusted**				**Adjusted**			
	Coef.	95% CI	SD	p-value	Coef.	95% CI	SD	p-value
Time	0.02	-0.04, 0.07	0.03	0.5458	0.06	0.00, 0.11	0.03	0.0606
High Comfort Distance (km)	-0.05	-0.14, 0.04	0.05	0.2782	-0.04	-0.13, 0.05	0.05	0.3979
Medium Comfort Distance (km)	0.02	-0.11, 0.15	0.07	0.7844	0.02	-0.11, 0.15	0.07	0.7245
Low Comfort Distance (km)	-0.03	-0.13, 0.05	0.05	0.3585	-0.03	-0.12, 0.07	0.05	0.5852
Baseline age					0.01	-0.01, 0.02	0.01	0.4469
Health Utility Index					0.86	-0.41, 2.13	0.65	0.1853
Education					-0.28	-0.65, 0.08	0.18	0.1272
Walkability Index					0.11	0.03, 0.18	0.04	0.0060
Immigrant					0.38	-0.17, 0.92	0.27	0.1736
Work/School					-0.42	-0.76, -0.09	0.17	0.0131
Marginalization Index					-0.11	-0.30, 0.07	0.09	0.2270
Movers					0.09	-0.20, 0.37	0.15	0.5547
Spring season					-0.30	-0.67, 0.07	0.19	0.1149
Summer season					-0.13	-0.46, 0.20	0.17	0.4426
Winter season					-0.07	-0.55, 0.41	0.24	0.7804

Random effects (adjusted model): Random intercept SD = 1.06, random slope SD = 0.19.

CI = confidence interval, SD = standard deviation
