# Supplementary material for: Cycling infrastructure as a determinant of cycling for recreation and transportation in Montréal, Canada: a natural experiment using the longitudinal national population health survey
Source: Int J Behav Nutr Phys Act. 2025 Jun 10;22:71. doi: 10.1186/s12966-025-01767-y (PMC12153112; doi:10.1186/s12966-025-01767-y)
Supplement: Supplementary file 4 — Supplementary Material 4 [file 12966_2025_1767_MOESM15_ESM.pdf]

Supplementary material 15. Associations between shortest distance to time varying type of cycling infrastructure from centroid of dissemination area and minutes per week of recreational cycling in men (N=187)

**Fixed Effects**	**Unadjusted**				**Adjusted**			
	Coef.	95% CI	SD	p-value	Coef.	95% CI	SD	p-value
Time	-0.01	-0.06, 0.04	0.03	0.8201	0.00	-0.05, 0.04	0.03	0.9907
High Comfort Distance (km)	0.00	-0.05, 0.06	0.03	0.9134	0.00	-0.06, 0.05	0.03	0.9415
Medium Comfort Distance (km)	0.01	-0.08, 0.10	0.04	0.7794	0.02	-0.07, 0.11	0.05	0.6195
Low Comfort Distance (km)	-0.05	-0.13, 0.03	0.04	0.2464	-0.02	-0.11, 0.07	0.04	0.6661
Baseline age					0.00	-0.01, 0.02	0.01	0.8316
Health Utility Index					0.58	-0.32, 1.48	0.46	0.2050
Education					0.09	-0.28, 0.47	0.19	0.6173
Walkability Index					0.06	-0.01, 0.14	0.04	0.0829
Immigrant					0.35	-0.12, 0.82	0.24	0.1443
Work/School					-0.23	-0.59, 0.14	0.19	0.2225
Marginalization Index					-0.11	-0.31, 0.10	0.10	0.3053
Movers					0.29	0.01, 0.58	0.13	0.0406
Spring season					-0.26	-0.64, 0.13	0.20	0.1895
Summer season					0.13	-0.21, 0.47	0.17	0.4611
Winter season					-0.45	-0.92, 0.02	0.24	0.0587

Random effects (adjusted model): Random intercept SD = 1.15, random slope SD = 0.15.

CI = confidence interval, SD = standard deviation
