# Supplementary material for: Cycling infrastructure as a determinant of cycling for recreation and transportation in Montréal, Canada: a natural experiment using the longitudinal national population health survey
Source: Int J Behav Nutr Phys Act. 2025 Jun 10;22:71. doi: 10.1186/s12966-025-01767-y (PMC12153112; doi:10.1186/s12966-025-01767-y)
Supplement: Supplementary file 5 — Supplementary Material 5 [file 12966_2025_1767_MOESM1_ESM.pdf]

Supplementary materials #1

Data cleaning and application of the Can-BICS

Prior to conducting analysis with the cycling data, we opted to perform inspections of patterns within the data to ensure its validity. We first observed that several infrastructure were changing back and forth between being existent and absent from one year to another (e.g., a bike lane is reported in 1991, 2001 and 2011, but not 1996 nor 2006). Given that it is unlikely that infrastructure would be constantly put in place and removed, we elected to fill in the years without any infrastructure reported for a street segment with the value of the period before only if infrastructure was reported in the period right after as well (e.g., if in 1996 no infrastructure were reported, but low-comfort infrastructure was reported in 2001 and high-comfort infrastructure was reported in 2001, then 2001 was classified as having low-comfort infrastructure).

Another potential issue we observed during our initial verification of the data in R, was that several infrastructure were changing from high comfort to medium or low comfort from one period to the next. While such reality is possible, it remains very unlikely primarily given that the classification of high comfort cycling infrastructure in the Can-BICS requires physical separation, which are not always simple to remove (aside from bollards). As such, we elected to perform visual verifications of reported cycling infrastructure for 2006 and 2011 using google street view for segments that had been flagged with the previously discussed issue.

After a first round of verification, we found that a majority of segments were incorrectly classified for at least 2006 or 2011. Three primary systematic issues were observed for the method cycling typologies were converted to the Can-BICS classification. First, infrastructure categorized as “bike lanes”, turned out to be sharrows, which do not conform to the Can-BICS classification, as was the case in previous studies that converted municipal typologies to the Can-BICS (1). A second consistent issue observed was the inclusion of all park paths within the medium comfort level of the Can-BICS, including unpaved paths which are non-conforming to the Can-BICS classification. A last recurrent issue was the categorization of cycling infrastructure physically separated from the road with bollards. Such infrastructure were almost always categorized as “physically-separated infrastructure” with the municipal typology and thus directly converted to the high level of comfort of the Can-BICS. However, the Can-Bics provide a strict maximum of 6 meters between bollards for such infrastructure to be considered high comfort, with such threshold not being met for the majority of cycling infrastructure that had bollards.

Given these issues, we first excluded all infrastructure classified as “bike lanes” in the municipal typology (Type 4) from the Can-BICS classification. We then expanded the visual verification of the segments using google street view to all street segments with infrastructure reported in either 2006 or 2011. When a segment was improperly classified for 2006 and 2011, we applied the rectification backwards to other years if they had the

same original classification as the one replaced in either 2006 or 2011. Summary data of the composition of the data before and after the rectification to apply the Can-BICs protocol are presented in Table 1 below.

Table 1 Number of segment and total length of infrastructure per Can-BICS comfort level per year before and after manual verifications of the data

		Number of segments		Total length (km)	
**Year**	**Can-BICS**	**Before**	**After**	**Before**	**After**
**1991**	Low	1898	973	224	106
	Medium	959	836	237	179
	High	295	174	34	28
**1996**	Low	2203	1215	264	137
	Medium	1131	1013	286	225
	High	349	153	44	24
**2001**	Low	2854	1685	316	179
	Medium	1476	1289	335	265
	High	518	233	72	36
**2006**	Low	2860	1868	321	198
	Medium	1798	1577	397	326
	High	1043	444	131	54
**2011**	Low	2468	2651	281	282
	Medium	2390	1978	503	393
	High	2330	616	255	75

REFERENCES

1. Ferster C, Fischer J, Manaugh K, Nelson T, Winters M. Using OpenStreetMap to inventory bicycle infrastructure: A comparison with open data from cities. International Journal of Sustainable Transportation. 2020;14(1):64-73.
