# Supplementary material for: Cycling infrastructure as a determinant of cycling for recreation and transportation in Montréal, Canada: a natural experiment using the longitudinal national population health survey
Source: Int J Behav Nutr Phys Act. 2025 Jun 10;22:71. doi: 10.1186/s12966-025-01767-y (PMC12153112; doi:10.1186/s12966-025-01767-y)
Supplement: Supplementary file 6 — Supplementary Material 6 [file 12966_2025_1767_MOESM2_ESM.pdf]

Supplementary materials 2 - Model specification

Let Y_{ij} be participant i's cycling response at the jth time point, i=1,...,n, j=1,...,n_i (note that participants can have different numbers of measurements due to dropout, noncompliance, different start dates, etc.). Let $\boldsymbol{X_{ij}}$ be subject i's covariate vector at time point j, consisting of fixed and time-varying covariates of length p. Define $\pi_{ij}=P(Y_{ij}=1)$, the probability that participant i at time point j has cycled in the past 3 months for recreation or transport. The basic model specification of our mixed effects logistic regression models the log odds as a function of the linear predictor plus random effects,

$$Log(\pi_{ij}/(1- \pi_{ij})) = \beta_0 + \beta_1 X_1 + ... + \beta_p X_p + b_{0,i} + b_{1,i} * j + \varepsilon_{ij}$$

Where $b_{0,i}$ and $b_{1,i}$ are the random intercept and slope respectively, and assumed to be normally distributed with mean 0 and variance-covariance matrix $\boldsymbol{\Sigma}$, N(0, $\boldsymbol{\Sigma}$). Additionally, €$_{ij}$ is the residual error term, also assumed to be Normally distributed with mean 0 and variance σ^2, N(0, σ^2). The residuals are assumed to be independent and identically distributed, and independent of the random effects.

If we define $\boldsymbol{X_{ij} = (1, X_1, ..., X_p)}$ and $\boldsymbol{Z_{ij} = (1, j)}$ to be the vector of fixed and random effects for subject i at time j, and $\mathbf{b_i}=(b_{0,i}, b_{1,i})$ the vector of random effects, we can succinctly write our model as: $Log(\pi_{ij}/(1- \pi_{ij})) = \boldsymbol{X_{ij}^T} * \boldsymbol{\beta} + \boldsymbol{Z_{ij}^T} * \mathbf{b_i} + \varepsilon_{ij.}$ Where $\boldsymbol{\beta}$ is the p_x1 vector $(\beta_0,\beta_1,...,\beta_p)$.

Similarly, if we let Wij be participant i's minutes of cycling per week at time point j, then our

linear mixed effects model is a quick replacement of the outcome variable, $\text{Log}(W_{ij}) = \boldsymbol{X}_{ij}^{T} * \boldsymbol{\beta} + \boldsymbol{Z}_{ij}^{T} * \mathbf{b}_i + \varepsilon_{ij}$. The fixed effects estimates can now be interpreted as the change in log minutes of cycling associated with a 1-unit increase in the respective covariate.
