# Supplementary material for: Cycling infrastructure as a determinant of cycling for recreation and transportation in Montréal, Canada: a natural experiment using the longitudinal national population health survey
Source: Int J Behav Nutr Phys Act. 2025 Jun 10;22:71. doi: 10.1186/s12966-025-01767-y (PMC12153112; doi:10.1186/s12966-025-01767-y)
Supplement: Supplementary file 7 — Supplementary Material 7 [file 12966_2025_1767_MOESM3_ESM.pdf]

Supplementary materials #3 – Attrition analysis

	C1 (overall)	C1 lost (not in C2)	Weighted t-test or chisq test p-val.
Unweighted # of subjects	600	20	
Weighted % cycle for recreation	22%	32%	0.543
Weighted % transport cycle	7%	16%	0.167
Weighted % any cycle	24%	32%	0.709
Weighted HUI score	0.89	0.89	0.984
Weighted Age	43	37	0.679

	C2 (overall)	C2 lost (not in C3)	Weighted t-test or chisq test p-val.
Unweighted # of subjects	611	94	
Weighted % cycle for recreation	21%	16%	0.161
Weighted % transport cycle	9%	9%	0.906
Weighted % any cycle	24%	20%	0.291
Weighted HUI score	0.92	0.85	**0.001**
Weighted Age	44	44	0.849

	C3 (overall)	C3 lost (not in C4)	Weighted t-test or chisq test p-val.
Unweighted # of subjects	559	76	

Weighted % cycle for recreation	24%	23%	0.878
Weighted % transport cycle	6%	3%	0.153
Weighted % any cycle	25%	23%	0.653
Weighted HUI score	0.92	0.89	0.296
Weighted Age	45	46	0.936

	C4 (overall)	C4 lost (not in C5)	Weighted t-test or chisq test p-val.
Unweighted # of subjects	545	92	
Weighted % cycle for recreation	19%	19%	0.806
Weighted % transport cycle	6%	5%	0.725
Weighted % any cycle	19%	29%	0.766
Weighted HUI score	0.92	0.80	0.090
Weighted Age	45	43	0.679

	C5 (overall)	C5 lost (not in C6)	Weighted t-test or chisq test p-val.
Unweighted # of subjects	495	93	
Weighted % cycle for recreation	24%	26%	0.607
Weighted % transport cycle	8%	7%	0.607

Weighted % any cycle	26%	26%	0.99
Weighted HUI score	0.91	0.90	0.38
Weighted Age	46	44	0.238

	C6 (overall)	C6 lost (not in C7)	Weighted t-test or chisq test p-val.
Unweighted # of subjects	454	70	
Weighted % cycle for recreation	21%	30%	0.067
Weighted % transport cycle	6%	11%	0.087
Weighted % any cycle	22%	30%	0.136
Weighted HUI score	0.90	0.88	0.2
Weighted Age	48	46	0.755

	C7 (overall)	C7 lost (not in C8)	Weighted t-test or chisq test p-val.
Unweighted # of subjects	456	76	
Weighted % cycle for recreation	29%	29%	0.981
Weighted % transport cycle	8%	11%	0.268
Weighted % any cycle	29%	29%	0.935
Weighted HUI score	0.88	0.87	0.095
Weighted Age	47	46	0.916

	C8 (overall)	C8 lost (not in C9)	Weighted t-test or chisq test p-val.
Unweighted # of subjects	423	62	
Weighted % cycle for recreation	21%	23%	0.862
Weighted % transport cycle	6%	5%	0.738
Weighted % any cycle	23%	23%	0.810
Weighted HUI score	0.88	0.80	0.056
Weighted Age	48	46	0.593
