# Supplementary material for: Cycling infrastructure as a determinant of cycling for recreation and transportation in Montréal, Canada: a natural experiment using the longitudinal national population health survey
Source: Int J Behav Nutr Phys Act. 2025 Jun 10;22:71. doi: 10.1186/s12966-025-01767-y (PMC12153112; doi:10.1186/s12966-025-01767-y)
Supplement: Supplementary file 8 — Supplementary Material 8 [file 12966_2025_1767_MOESM4_ESM.pdf]

Supplementary materials 4. Total annual length and change between 1991 and 2011 of cycling infrastructure in the Montreal CMA

Can-BICS category	Total length of cycling network (m)					Change in cycling infrastructure length from 1991 to 2011	
	1991	1996	2001	2006	2011	Meters	Km
Low comfort	106,266.2	137,338.0	178,962.2	197,765.5	281,890.0	+175,623.8	+175.6
Medium comfort	179,189.4	225,093.3	264,990.7	325,999.3	393,338.8	+214,149.4	+214.1
High comfort	28,103.2	24,207.4	36,022.2	53,832.7	75,485.0	+47,381.8	+47.4
Non-conforming	114,404.9	141,328.3	176,370.0	197,644.7	267,998.5	+153,593.6	+153.6
Total	427,963.7	527,967.0	656,345.1	775,242.2	1,018,712.3	+590,748.6	+590.7
