# Supplementary material for: Cycling infrastructure as a determinant of cycling for recreation and transportation in Montréal, Canada: a natural experiment using the longitudinal national population health survey
Source: Int J Behav Nutr Phys Act. 2025 Jun 10;22:71. doi: 10.1186/s12966-025-01767-y (PMC12153112; doi:10.1186/s12966-025-01767-y)
Supplement: Supplementary file 9 — Supplementary Material 9 [file 12966_2025_1767_MOESM5_ESM.pdf]

Supplementary material 5. Associations between access to cycling infrastructure within distance thresholds and any cycling in men (N=344)

**Fixed Effects**	**Unadjusted**				**Adjusted**			
	OR	95% CI	SD	p-value	OR	95% CI	SD	p-value
Time	0.96	0.90, 1.03	0.03	0.2203	0.91	0.85, 0.98	0.04	0.0091
High Comfort Threshold (<1790m)	1.15	0.83, 1.62	0.17	0.4016	1.24	0.88, 1.75	0.17	0.2110
Medium Comfort Threshold (<623m)	0.73	0.46, 1.15	0.23	0.1770	0.78	0.50, 1.24	0.23	0.3017
Low Comfort Threshold (<321m)	1.09	0.63, 1.87	0.28	0.7680	0.90	0.52, 1.58	0.28	0.7240
Baseline age					0.97	0.95, 0.98	0.01	0.0000
Health Utility Index					1.54	0.45, 5.29	0.63	0.4956
Education					1.37	0.83, 2.28	0.26	0.2208
Walkability Index					0.90	0.81, 0.99	0.05	0.0272
Immigrant					0.67	0.37, 1.21	0.30	0.1831
Work/School					0.94	0.59, 1.49	0.23	0.7931
Marginalization Index					0.83	0.64, 1.08	0.13	0.1716
Movers					0.65	0.45, 0.94	0.19	0.0223
Spring season					0.80	0.48, 1.34	0.26	0.3974
Summer season					1.63	1.01, 2.63	0.24	0.0471
Winter season					0.17	0.10, 0.29	0.28	0.0000

Random effects (adjusted model): Random intercept variance = 2.02, random slope variance = 0.08. CI = confidence interval, OR = odds ratio, SD = standard deviation
