# Supplementary material for: Cycling infrastructure as a determinant of cycling for recreation and transportation in Montréal, Canada: a natural experiment using the longitudinal national population health survey
Source: Int J Behav Nutr Phys Act. 2025 Jun 10;22:71. doi: 10.1186/s12966-025-01767-y (PMC12153112; doi:10.1186/s12966-025-01767-y)
Supplement: Supplementary file 10 — Supplementary Material 10 [file 12966_2025_1767_MOESM6_ESM.pdf]

Supplementary material 6. Associations between access to cycling infrastructure within distance thresholds and any cycling in women (N=432)

**Fixed Effects**	**Unadjusted**				**Adjusted**			
	OR	95% CI	SD	p-value	OR	95% CI	SD	p-value
Time	0.94	0.88, 0.995	0.03	0.0345	0.91	0.86, 0.97	0.03	0.0047
High Comfort Threshold (<1790m)	1.28	0.92, 1.77	0.17	0.1450	1.26	0.90, 1.78	0.18	0.1831
Medium Comfort Threshold (<623m)	0.84	0.55, 1.28	0.22	0.4194	0.94	0.61, 1.46	0.22	0.7917
Low Comfort Threshold (<321m)	1.55	0.94, 2.56	0.26	0.0846	1.35	0.79, 2.32	0.28	0.2777
Baseline age					0.96	0.95, 0.98	0.01	0.0000
Health Utility Index					4.27	1.14, 16.04	0.67	0.0314
Education					1.05	0.65, 1.70	0.25	0.8348
Walkability Index					1.05	0.96, 1.16	0.05	0.2886
Immigrant					0.58	0.32, 1.04	0.30	0.0691
Work/School					1.73	1.16, 2.60	0.21	0.0079
Marginalization Index					0.80	0.62, 1.02	0.13	0.0677
Movers					1.02	0.71, 1.46	0.19	0.9247
Spring season					0.65	0.40, 1.07	0.25	0.0896
Summer season					1.36	0.85, 2.16	0.24	0.1963
Winter season					0.16	0.09, 0.29	0.28	0.0000

Random effects (adjusted model): Random intercept variance = 1.55, random slope variance = 0.02. CI = confidence interval, OR = odds ratio, SD = standard deviation
