# Supplementary material for: Cycling infrastructure as a determinant of cycling for recreation and transportation in Montréal, Canada: a natural experiment using the longitudinal national population health survey
Source: Int J Behav Nutr Phys Act. 2025 Jun 10;22:71. doi: 10.1186/s12966-025-01767-y (PMC12153112; doi:10.1186/s12966-025-01767-y)
Supplement: Supplementary file 11 — Supplementary Material 11 [file 12966_2025_1767_MOESM7_ESM.pdf]

Supplementary material 7. Associations between cumulative years of exposure to cycling infrastructure within distance thresholds and any cycling in men (N=344)

**Fixed Effects**	**Unadjusted**				**Adjusted**			
	OR	95% CI	SD	p-value	OR	95% CI	SD	p-value
Time	1.11	0.96, 1.28	0.07	0.1608	0.98	0.84, 1.14	0.08	0.7624
High Comfort Threshold (<1790m)	**0.90**	**0.82, 0.98**	**0.05**	**0.0227**	0.93	0.84, 1.03	0.05	0.1480
Medium Comfort Threshold (<623m)	**0.89**	**0.78, 1.02**	**0.07**	**0.0978**	0.94	0.82, 1.09	0.07	0.4169
Low Comfort Threshold (<321m)	0.94	0.86, 1.01	0.04	0.1019	0.98	0.90, 1.07	0.04	0.6384
Baseline age					0.97	0.95, 0.98	0.01	0.0001
Health Utility Index					1.48	0.43, 5.09	0.63	0.5383
Education					1.39	0.83, 2.31	0.26	0.2130
Walkability Index					0.89	0.81, 0.98	0.05	0.0230
Immigrant					0.65	0.36, 1.18	0.30	0.1563
Work/School					0.94	0.59, 1.49	0.24	0.7789
Marginalization Index					0.86	0.66, 1.11	0.13	0.2541
Movers					0.64	0.44, 0.94	0.19	0.0209
Spring season					0.82	0.49, 1.38	0.27	0.4575
Summer season					1.68	1.04, 2.74	0.25	0.0355
Winter season					0.17	0.10, 0.30	0.29	0.0000

Random effects (adjusted model): Random intercept variance = 2.15, random slope variance = 0.08. CI = confidence interval, OR = odds ratio, SD = standard deviation
