# Supplementary material for: Cycling infrastructure as a determinant of cycling for recreation and transportation in Montréal, Canada: a natural experiment using the longitudinal national population health survey
Source: Int J Behav Nutr Phys Act. 2025 Jun 10;22:71. doi: 10.1186/s12966-025-01767-y (PMC12153112; doi:10.1186/s12966-025-01767-y)
Supplement: Supplementary file 12 — Supplementary Material 12 [file 12966_2025_1767_MOESM8_ESM.pdf]

Supplementary material 8. Associations between cumulative years of exposure to cycling infrastructure within distance thresholds and any cycling in women (N=432)

Fixed Effects	Unadjusted				Adjusted			
	OR	95% CI	SD	p-value	OR	95% CI	SD	p-value
Time	1.17	1.00, 1.36	0.08	0.0471	0.93	0.79, 1.09	0.08	0.3814
High Comfort Threshold (<1790m)	**0.92**	**0.84, 1.01**	**0.05**	**0.0832**	1.03	0.94, 1.14	0.05	0.4993
Medium Comfort Threshold (<623m)	**0.86**	**0.76, 0.98**	**0.07**	**0.0213**	0.98	0.85, 1.12	0.07	0.7544
Low Comfort Threshold (<321m)	**0.87**	**0.80, 0.95**	**0.04**	**0.0015**	0.97	0.89, 1.06	0.05	0.5166
Baseline age					0.96	0.94, 0.98	0.01	0.0000
Health Utility Index					4.36	1.17, 16.28	0.67	0.0283
Education					1.02	0.63, 1.65	0.24	0.9339
Walkability Index					1.07	0.97, 1.17	0.05	0.1884
Immigrant					0.57	0.32, 1.04	0.30	0.0687
Work/School					1.70	1.13, 2.56	0.21	0.0104
Marginalization Index					0.79	0.62, 1.01	0.12	0.0646
Movers					1.02	0.71, 1.47	0.19	0.9114
Spring season					0.68	0.42, 1.11	0.25	0.1257
Summer season					1.39	0.87, 2.21	0.24	0.1635
Winter season					0.16	0.09, 0.28	0.28	0.0000

Random effects (adjusted model): Random intercept variance = 1.54, random slope variance = 0.02. CI = confidence interval, OR = odds ratio, SD = standard deviation
