# Supplementary material for: Cycling infrastructure as a determinant of cycling for recreation and transportation in Montréal, Canada: a natural experiment using the longitudinal national population health survey
Source: Int J Behav Nutr Phys Act. 2025 Jun 10;22:71. doi: 10.1186/s12966-025-01767-y (PMC12153112; doi:10.1186/s12966-025-01767-y)
Supplement: Supplementary file 13 — Supplementary Material 13 [file 12966_2025_1767_MOESM9_ESM.pdf]

Supplementary material 9. Associations between shortest distance to time varying type of cycling infrastructure from centroid of dissemination area and any cycling in men (N=344)

Fixed Effects	Unadjusted				Adjusted			
	OR	95% CI	SD	p-value	OR	95% CI	SD	p-value
Time	0.97	0.91, 1.04	0.03	0.3827	0.92	0.85, 0.99	0.04	0.0189
High Comfort Distance (km)	1.00	0.91, 1.09	0.05	0.9639	0.99	0.90, 1.08	0.05	0.7583
Medium Comfort Distance (km)	1.08	0.93, 1.25	0.08	0.2896	1.04	0.90, 1.21	0.07	0.5851
Low Comfort Distance (km)	1.03	0.91, 1.17	0.06	0.6042	1.03	0.91, 1.17	0.06	0.6094
Baseline age					0.96	0.95, 0.98	0.01	0.0000
Health Utility Index					1.48	0.43, 5.11	0.63	0.5310
Education					1.34	0.81, 2.24	0.26	0.2547
Walkability Index					0.90	0.82, 1.00	0.05	0.0431
Immigrant					0.65	0.36, 1.17	0.30	0.1473
Work/School					0.94	0.59, 1.48	0.24	0.7790
Marginalization Index					0.85	0.65, 1.12	0.14	0.2430
Movers					0.65	0.44, 0.94	0.19	0.0223
Spring season					0.78	0.47, 1.31	0.26	0.3489
Summer season					1.60	0.99, 2.60	0.25	0.0556
Winter season					0.17	0.10, 0.29	0.28	0.0000

Random effects (adjusted model): Random intercept variance = 2.10, random slope variance = 0.08. CI = confidence interval, OR = odds ratio, SD = standard deviation
