# Supplementary material for: Cycling infrastructure as a determinant of cycling for recreation and transportation in Montréal, Canada: a natural experiment using the longitudinal national population health survey
Source: Int J Behav Nutr Phys Act. 2025 Jun 10;22:71. doi: 10.1186/s12966-025-01767-y (PMC12153112; doi:10.1186/s12966-025-01767-y)
Supplement: Supplementary file 14 — Supplementary Material 14 [file 12966_2025_1767_MOESM10_ESM.pdf]

Supplementary material 10. Associations between shortest distance to type of time varying cycling infrastructure from centroid of dissemination area and any cycling in women (N=432)

Fixed Effects	Unadjusted				Adjusted			
	OR	95% CI	SD	p-value	OR	95% CI	SD	p-value
Time	0.94	0.89, 1.00	0.03	0.0511	0.91	0.85, 0.97	0.03	0.0030
High Comfort Distance (km)	0.92	0.83, 1.02	0.05	0.1193	0.92	0.83, 1.02	0.05	0.1033
Medium Comfort Distance (km)	1.08	0.92, 1.26	0.08	0.3670	1.02	0.88, 1.20	0.08	0.7646
Low Comfort Distance (km)	0.99	0.88, 1.11	0.06	0.8082	0.99	0.87, 1.11	0.06	0.8160
Baseline age					0.96	0.95, 0.98	0.01	0.0000
Health Utility Index					4.30	1.14, 16.18	0.68	0.0309
Education					1.03	0.64, 1.67	0.24	0.8938
Walkability Index					1.05	0.95, 1.15	0.05	0.3785
Immigrant					0.56	0.31, 1.02	0.30	0.0561
Work/School					1.76	1.17, 2.64	0.21	0.0066
Marginalization Index					0.78	0.61, 0.99	0.13	0.0423
Movers					1.01	0.70, 1.45	0.19	0.9564
Spring season					0.68	0.42, 1.11	0.25	0.1236
Summer season					1.40	0.88, 2.22	0.24	0.1512
Winter season					0.17	0.10, 0.29	0.28	0.0000

Random effects (adjusted model): Random intercept variance = 1.55, random slope variance = 0.02. CI = confidence interval, OR = odds ratio, SD = standard deviation
