# Supplementary material for: Cycling infrastructure as a determinant of cycling for recreation and transportation in Montréal, Canada: a natural experiment using the longitudinal national population health survey
Source: Int J Behav Nutr Phys Act. 2025 Jun 10;22:71. doi: 10.1186/s12966-025-01767-y (PMC12153112; doi:10.1186/s12966-025-01767-y)
Supplement: Supplementary file 15 — Supplementary Material 15 [file 12966_2025_1767_MOESM11_ESM.pdf]

Supplementary material 11. Associations between access to cycling infrastructure within distance thresholds and log minutes per week of recreational cycling in men (N=187)

**Fixed Effects**	**Unadjusted**				**Adjusted**			
	Coef.	95% CI	SD	p-value	Coef.	95% CI	SD	p-value
Time	0.00	-0.05, 0.05	0.03	0.8664	0.01	-0.05, 0.06	0.03	0.8184
High Comfort Threshold (<1790m)	-0.21	-0.46, 0.04	0.13	0.0931	-0.20	-0.47, 0.06	0.13	0.1383
Medium Comfort Threshold (<623m)	-0.16	-0.51, 0.19	0.18	0.3643	-0.18	-0.54, 0.18	0.18	0.3202
Low Comfort Threshold (<321m)	0.13	-0.29, 0.56	0.22	0.5454	0.07	-0.38, 0.51	0.23	0.7656
Baseline age					0.00	-0.01, 0.01	0.01	0.9371
Health Utility Index					0.51	-0.40, 1.41	0.46	0.2720
Education					0.09	-0.29, 0.47	0.20	0.6433
Walkability Index					0.07	0.00, 0.14	0.04	0.0548
Immigrant					0.33	-0.15, 0.80	0.24	0.1809
Work/School					-0.25	-0.61, 0.12	0.19	0.1876
Marginalization Index					-0.08	-0.28, 0.12	0.10	0.4362
Movers					0.27	-0.01, 0.55	0.14	0.0631
Spring season					-0.27	-0.66, 0.12	0.20	0.1757
Summer season					0.08	-0.26, 0.43	0.18	0.6375
Winter season					-0.47	-0.94, 0.00	0.24	0.0505

Random effects (adjusted model): Random intercept variance = 1.12, random slope variance = 0.15. CI = confidence interval, SD = standard deviation
