# Supplementary material for: Cycling infrastructure as a determinant of cycling for recreation and transportation in Montréal, Canada: a natural experiment using the longitudinal national population health survey
Source: Int J Behav Nutr Phys Act. 2025 Jun 10;22:71. doi: 10.1186/s12966-025-01767-y (PMC12153112; doi:10.1186/s12966-025-01767-y)
Supplement: Supplementary file 16 — Supplementary Material 16 [file 12966_2025_1767_MOESM12_ESM.pdf]

Supplementary material 12. Associations between access to cycling infrastructure within distance thresholds and log minutes per week of recreational cycling in women (N=192)

**Fixed Effects**	**Unadjusted**				**Adjusted**			
	Coef.	95% CI	SD	p-value	Coef.	95% CI	SD	p-value
Time	0.02	-0.03, 0.08	0.03	0.4269	0.06	0.00, 0.11	0.03	0.0504
High Comfort Threshold (<1790m)	0.15	-0.12, 0.43	0.14	0.2694	0.16	-0.12, 0.43	0.14	0.2630
Medium Comfort Threshold (<623m)	-0.02	-0.34, 0.30	0.16	0.9011	-0.08	-0.40, 0.25	0.17	0.6491
Low Comfort Threshold (<321m)	-0.05	-0.45, 0.35	0.20	0.8002	-0.01	-0.43, 0.40	0.21	0.9507
Baseline age					0.01	-0.01, 0.02	0.01	0.4251
Health Utility Index					0.79	-0.49, 2.06	0.65	0.2250
Education					-0.28	-0.64, 0.09	0.19	0.1424
Walkability Index					0.12	0.04, 0.19	0.04	0.0020
Immigrant					0.40	-0.15, 0.95	0.29	0.1555
Work/School					-0.43	-0.77, -0.10	0.17	0.0115
Marginalization Index					-0.11	-0.30, 0.07	0.09	0.2381
Movers					0.09	-0.20, 0.37	0.14	0.5390
Spring season					-0.30	-0.67, 0.07	0.19	0.1160
Summer season					-0.14	-0.47, 0.19	0.17	0.4146
Winter season					-0.08	-0.56, 0.40	0.24	0.7377

Random effects (adjusted model): Random intercept SD = 1.07, random slope SD = 0.19.

CI = confidence interval, SD = standard deviation
